# The Association between ER, PR, HER2, and ER−/PR+ Expression and Lung Cancer Subsequent in Breast Cancer Patients: A Retrospective Cohort Study Based on SEER Database

**DOI:** 10.1155/2023/7028189

**Published:** 2023-11-11

**Authors:** Hong Yu Shao, Bao Tan Hao, Feng Xiao Gao

**Affiliations:** ^1^Department of CT/MR, Xingtai People's Hospital, Xingtai, Hebei, China; ^2^Department of Thoracic Surgery, Xingtai People's Hospital, Xingtai, Hebei, China

## Abstract

**Aims:**

The available research on the association between estrogen receptor (ER), progesterone receptor (PR), human epidermal growth factor receptor 2 (HER2), ER−/PR+ status, and the occurrence of lung cancer subsequent to breast cancer in patients (referred to as BC-LuC) had been limited. Consequently, there is a need to examine whether ER, PR, HER2, and ER−/PR+ have independent correlations with the risk and outcomes of BC-LuC, while appropriately adjusting for other potential covariates.

**Methods:**

The present study employed a cohort design and utilized data from the Surveillance, Epidemiology, and End Results (SEER) program spanning from 2010 to 2015. The study population consisted of 683,336 individuals who were diagnosed with breast cancer (referred to as BC). Various covariates were assessed at baseline, including age, sex, race, marital status, CS tumor size, laterality, radiation, chemotherapy, months from diagnosis to treatment, breast subtype, AJCC 7th edition (2010–2015), and combined summary stage (2004+). The primary objective of this study was to investigate the association between ER, PR, HER2, ER−/PR+ status, and the risk of developing BC-LuC. Logistic regression analysis was employed to assess this association. Furthermore, multivariable Cox regression analyses were conducted to calculate adjusted hazard ratios (HRs) along with their respective 95% confidence intervals (CIs). Kaplan–Meier plots and log-rank tests were utilized to estimate the outcomes, specifically overall survival (OS), disease-specific survival (DSS), and metastasis.

**Results:**

The average age of 198,972 selected participants was 59.8 ± 13.1 years, and about 99.3% of them were female. Result of fully adjusted binary logistic regression showed PR+ and HER2+ were positively associated with lower risk BC-LuC after adjusting confounders (ORs = 0.84, 95% CI: 0.73–0.96, *p* = 0.011 and ORs = 0.83, 95% CI: 0.72–0.96, *p* = 0.012, respectively). ER+ and ER−/PR+ were detected no significant relationship with BC-LuC (ORs = 1.03, 95% CI: 0.87–1.22, *p* = 0.718 and ORs = 1.02, 95% CI: 0.61–1.72, *p* = 0.936, respectively). In subgroups analyses, the results remain stable. Multivariable Cox regression showed that BC-LuC patients with ER and PR were significantly associated with OS and DSS. However, ER, PR, HER2, and ER−/PR+ were significantly associated with OS and DSS in breast cancer patients. The relationship between ER, PR, HER2, and ER−/PR+ and metastasis in breast cancer patients was different.

**Conclusion:**

The results of this study indicated a potential correlation between PR- and HER2- status and a risk of developing BC-LuC. Furthermore, it appears that the prognosis of BC-LuC may be influenced by the presence of ER+ and PR+. Therefore, additional research is warranted to fully investigate and validate this association.

## 1. Introduction

Breast cancer has the first highest cancer incidence and mortality rates among women, posing a significant public health challenge and burden in both the United States and China [1, 2]. However, advancements in screening modalities, such as molybdenum target X-ray and color Doppler ultrasound, coupled with improvements in diagnosis, have contributed to an increasing number of breast cancer survivors. Numerous studies had documented a heightened risk of developing a second primary lung cancer among individuals who have overcome an initial breast cancer diagnosis [3, 4]. Notably, approximately ten percent of breast cancer patients who surpass the ten-year survival threshold are afflicted by a subsequent primary carcinoma, with lung cancer being a predominant manifestation. The emergence of a second primary lung cancer can complicate clinical assessments and necessitate aggressive interventions, further amplifying the burdens faced by breast cancer survivors.

Accurate assessment of the steroid hormone receptor status, particularly estrogen and progesterone receptors (ER and PR), is essential for effective management of invasive breast cancer. The HR status is a critical determinant of prognosis and treatment strategies for breast cancer. Currently, immunohistochemistry (IHC) represents the best method for detecting ER and PR status [5]. Given the clinical, morphological, and molecular heterogeneity of breast cancer, a wide range of variations in its treatment and management may occur [6]. Notably, the St. Gallen surrogated for breast cancer subtypes has identified five primary entities based on the molecular classification through immunohistochemical expression of ER, PR, HER2, and proliferation index Ki-67: luminal-A, luminal-B, HER2-negative, luminal-B HER2-positive, HER2 enriched, and TNBC (triple negative: lack of expression of ER, PR, and no overexpression of HER2) [7, 8]. While some studies had categorized ER−/PR+ breast cancer as technical artifacts in IHC, previous research illustrated that this is a unique subtype with distinct molecular and clinical features [9]. Additionally, Gamrani et al.'s study [10] demonstrated a statistically significant association between the ER−/PR+ group and a higher risk of recurrence and death, placing it midway between the double-negative and double-positive HR subtypes. After endocrine therapy in breast cancer patients, those with ER−/PR+ exhibited higher disease-free survival rates and lower all-cause mortality than a modified group without endocrine treatment [11]. In a meta-analysis of early-stage breast cancer patients treated with endocrine therapy, ER+ breast cancer patients did not appear to benefit from endocrine treatment [12].

However, findings from previous studies regarding the relationship between ER, PR, HER2, and ER−/PR+ and lung cancer subsequent in breast cancer patients was limited. In this study, we aimed to explore the relationship between ER, PR, HER2, and ER−/PR+ and the risk and outcomes of BC-LuC. To this end, we analyzed 198,972 breast cancer patients which had been previously diagnosed in SEER database from 2010 to 1015.

## 2. Participants and Methods

### 2.1. Database

A retrospective analysis was conducted utilizing the Surveillance, Epidemiology, and End Results (SEER) database, which was publicly released in November 2021. The study population consisted of over 600,000 breast cancer patients diagnosed between 2010 and 2015, providing a comprehensive representation of real-world data. Hongyu Shao, as one of the authors, obtained appropriate authorization (certification number 15391-Nov2021) to access and analyze the SEER database. Notably, institutional review board approval was not necessary for this particular study.

### 2.2. Study Population

In this study, cancers were defined based on the SEER variable site recode and associated ICD-O-3 values, which enabled the determination of the sequence and temporal aspects of all cancer diagnosis. The methodology employed in this regard has been described comprehensively in previous literature [13]. Initially, a cohort of 198,972 patients were identified for inclusion in the study based on the following predefined criteria: (1) age greater than or equal to 18 years, (2) diagnosed within the time period spanning from 2010 to 2015, and (3) received pathological confirmation of both breast cancer and lung cancer. Patients with a prior diagnosis of a third type of cancer preceding the occurrence of lung cancer, as well as those with incomplete datasets, were excluded from the analysis.

### 2.3. Covariates

We used covariates which was based on the previous studies as follows: the year of diagnosis, age (≥18 years old), sex (female and male), race (American Indian/Alaska Native, Asian/Pacific Islander, Black, and White), marital status (married, single, divorced, separated, and widowed), clinical staging(CS) tumor size, laterality (left, right, and bilateral), radiation, chemotherapy, months from diagnosis to treatment and breast subtype (luminal-A, luminal-B, HER2 enriched, and triple-negative). We described breast cancer stage using AJCC, 7th edition (2010–2015) and combined summary stage (2004+).

### 2.4. Outcomes

We measured three outcomes which were OS and DSS, and metastasis (bone, brain, liver, and lung). OS was the interval in months between breast cancer diagnosis and death from any cause. DSS was the interval in months between breast cancer diagnosis and death from breast cancer. Patients were followed until death or December 31, 2015.

### 2.5. Statistical Analysis

Data were categorized into continuous and categorical variables. Continuous variables were further divided into two types based on the normality of their distribution. Normally distributed continuous variables were presented as the mean ± standard deviation and compared between groups using the Student's t-test. Categorical variables were presented as percentages. Variables were compared using the Chi-square tests (categorical variables), one-way ANOVA (normal distribution), and Kruskal–Wallis (skewed distribution) test.

Logistic and multivariable Cox regression analyses were adopted to assess the ER, PR, HER2, and ER−/PR+ on the risk and outcomes in BC-LuC. An extended logistic model approach was used for different covariates adjusted models. Subgroup analyses were stratified by some relevant effect covariates. Survival curves were plotted by Kaplan–Meier and log-rank analyses. All the analyses were performed with the statistical software packages R 3.3.2 (https://www.R-project.org, The R Foundation) and free statistics software versions 1.5. A two-tailed test was performed and *p* < 0.05 was considered statistically significant.

## 3. Results

### 3.1. Baseline Characteristics

**Figure 1:**
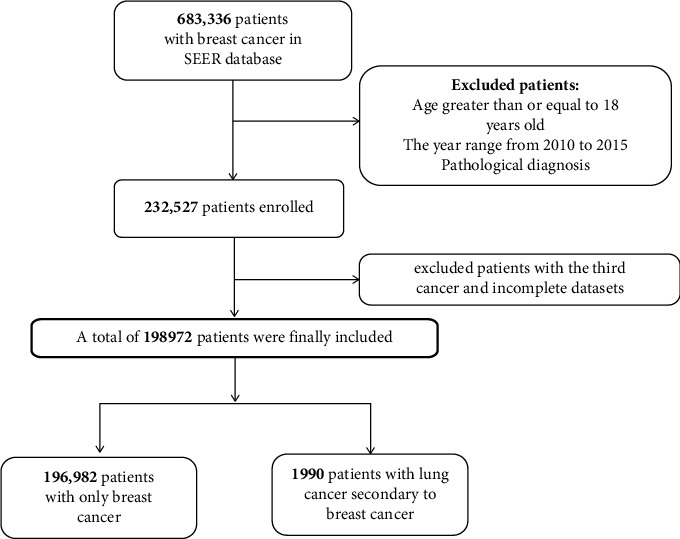
The flowchart of the study.

A total of 198.972 participants were included in the final analysis, as depicted in Figure 1. Baseline characteristics of these participants were presented in Table 1, stratified by the presence or absence of ER+,PR+,HER+ and ER−/PR+ status. On average, the selected participants had a mean age of 59.8 ± 13.1 years, with age categorized into three groups based on cutoffs of 40 and 60 years. Approximately, 99.3% of the participants were female. The prevalence of BC-LuC was comparably distributed across the four groups. Notably, patients aged below 60 years exhibited higher rates of HER2+ and ER−/PR+ subtypes, while the ER−/PR+ subtype was more prevalent among black individuals compared to other racial groups. The interval from diagnosis to treatment was categorized into three groups: ≤1 month, 1–3 months, and >3 months, with approximately 75.6% of the patients falling into the ≤1 month category, with approximately 75.6% of the patients falling into the ≤1 month category. Furthermore, an association was observed between larger tumor sizes (as per the CS) and increased occurrence of HER2+ and ER−/PR+ subtypes. Radiotherapy usage was comparable across all groups, although chemotherapy utilization was relatively higher among HER2+ and ER−/PR+ patients. Analysis of DSS and OS revealed a higher risk of mortality among individuals exhibiting ER−/PR+ status within the selected participant cohort.

### 3.2. Relationship between ER, PR, HER2, and ER−/PR+ and the Risk of BC-LuC

The results of our study are summarized in Table 2. In this investigation, we employed multivariate binary logistic regression to construct four models aimed at examining the independent effects of ER,PR,HER2 and ER−/PR+ status on the risk of BC-LuC. The odds ratios (ORs) along with their corresponding 95% confidence intervals (CIs) are presented in Table 2.

**Figure 2:**
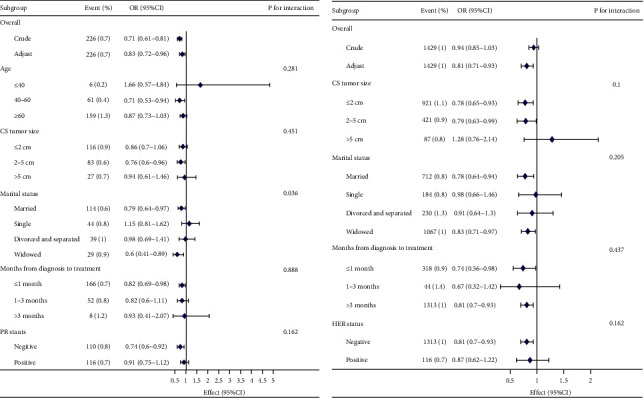
Association between HER2 (a) and PR (b) and risk of BC-LuC according to baseline characteristics. Each stratification adjusted for all the factors (age, sex, race, laterality, martial and month from diagnosis treatment AJCC, stage, CS tumor size, radiation and chemotherapy, and breast subtype).

In the unadjusted model, the observed effect size can be interpreted as the change in the risk of BC-LuC associated with a one-unit decrease in the presence of ER+, PR+, HER2+, or ER−/PR+. Specifically, for HER2+ patients with BC-LuC, the unadjusted model indicated a 29% reduction in risk (OR = 0.71, 95% CI: 0.61–0.81, *p* < 0.001, Table 2, Figure 2).

Following adjustment for all covariates, a 19% decreased risk was noted among PR+ patients with BC-LuC (HR = 0.81, 95% CI: 0.71–0.93, *p* = 0.002), while HER2+ patients exhibited a 17% lower risk (HR = 0.83, 95% CI: 0.72–0.96, *p* = 0.012, Table 2).

### 3.3. Subgroup Analysis

In order to observe the trend of effect sizes in various variables, namely, CS tumor size, marital status, months from diagnosis to treatment, PR+ status, and HER2+ status, we employed these variables as stratification factors (Figure 2). We assessed for potential interactions within subgroups but did not find any statistically significant interactions, except for the subgroup of individuals with HER2+ status in relation to marital status (*p*>0.05 for all other subgroups).

### 3.4. The Results of Outcomes of ER, PR, HER2, and ER−/PR+ in BC-LuC and BC

**Figure 3:**
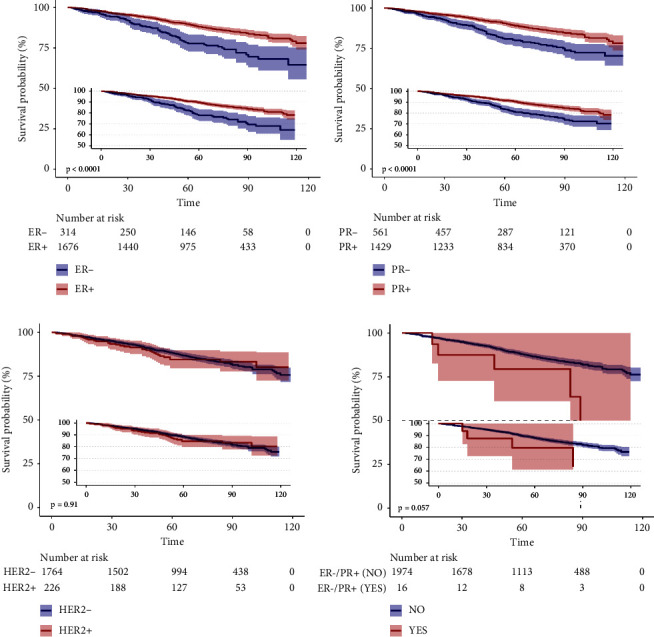
Kaplan–Meier survival curves for DSS of ER, PR, HER2, and ER−/PR+ in BC-LuC.

**Figure 4:**
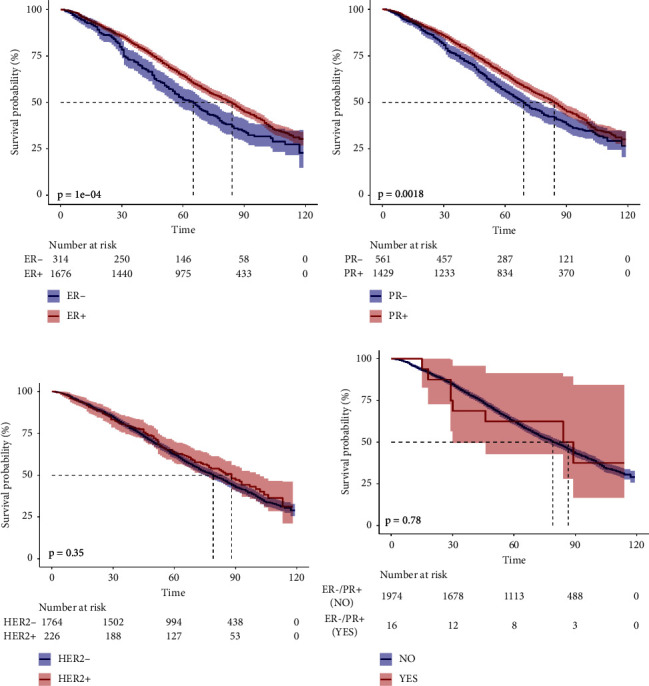
Kaplan–Meier survival curves for OS of ER, PR, HER2, and ER−/PR+ in BC-LuC.

In this study, we conducted an analysis to investigate the association between ER,PR,HER2, ER−/PR+ status, and various clinical outcomes including DSS,OS, and the occurrence of metastases in bone, brain, liver, and lung. Utilizing multivariable Cox models, we identified significant associations between the hazard ratios (HRs) of ER+ and PR2+ in relation to BC-LuC for both DSS and OS (Supplementary Table 1 and Figures 3 and 4 in the attachments). Furthermore, we observed that BC patients in the ER+, PR+, and HER2+ subgroups exhibited longer DSS and OS (Supplementary Table 2 and Supplementary Figures 1 and 2 in the attachments). In contrast, individuals with ER−/PR+ status in BC demonstrated lower disease-specific DSS and OS (Supplementary Table 2 and Supplementary Figures 1 and 2 in the attachments).

Due to the limited number of metastatic patients specifically within the BC-LuC subgroup, we conducted our analysis on the entire study population, which included both individuals with BCand BC-LuC. Through the utilization of multivariate binary logistic regression and subgroup analysis (Supplemetary Table 3 and 4 in the attachments), we explored the relationship between ER,PR,HER2 and ER−/PR+ status, and the occurrence of metastases in the bone, brain, liver, and lung.

Regarding bone metastasis, our findings revealed that HER2+ and ER−/PR+ status were associated with a reduced risk of metastasis, whereas ER+ and PR+ status exhibited an opposite effect. In the case of lung metastasis, ER+ status was found to be associated with a reduced risk, while no significant relationships were observed for the other groups. In terms of liver metastasis, both ER2+ and PR+ status were associated with a reduced risk, whereas HER2+ and ER−/PR+ status manifested an opposite effect. Lastly, for brain metastasis, our analysis demonstrated that ER+ and PR+ status were associated with a reduced risk, whereas no significant associations were observed for the other groups.

## 4. Discussion

Our findings revealed a significant positive association between PR+ and HER2+ status and a lower risk of BC-LuC when adjusting for other covariates. Furthermore, the results of our study identified stronger associations in CS tumor size, months from diagnosis to treatment, PR+ status, and HER2+ status. The use of subgroup analysis allowed for a more comprehensive understanding of the trend towards a lower risk of BC-LuC in different populations. Moreover, our study demonstrated that ER+ and PR+ status were associated with increased DSS and OS in BC-LuC. Similarly, ER+, PR+, and HER2+ status were associated with improved DSS and OS in BC cases. Conversely, an opposite effect was observed in BC cases with ER−/PR+ status in relation to DSS and OS. Moreover, our study demonstrated that ER+ and PR+ status were associated with increased DSS and OS in BC-LuC. Similarly, ER+, PR+, and HER2+ status were associated with improved DSS and OS in BC cases. Conversely, an opposite effect was observed in BC cases with ER−/PR+ status in relation to DSS and OS. Moreover, our study demonstrated that ER+ and PR+ status were associated with increased DSS and OS in BC-LuC. Similarly, ER+, PR+, and HER2+ status were associated with improved DSS and OS in BC cases. Conversely, an opposite effect was observed in BC cases with ER−/PR+ status in relation to DSS and OS.

As per the meta-analysis conducted by Wang, only one study investigated the relationship between the expression statuses of ER, PR, and HER2 and the risk of developing lung cancer subsequently in female patients with breast cancer [14]. In a study involving 535,941 breast cancer patients [15], the results indicated that a positive estrogen receptor status (RR = 0.93 and *p* = 0.014) and positive progesterone receptor status (RR = 0.86 and *p* < 0.001) were associated with a lower risk of developing BC-LuC. Another study conducted by Schonfeld et al. [16] demonstrated that the absence of any negative breast cancer receptor marker increased the risk of BC-LuC. Additionally, Lin's et al. study [17] reported that HER2 status had no significant effect on the development of BC-LuC. These conclusions align precisely with the findings of our own study. Several potential factors may account for the disparity in results observed between our study and previous research. First, variations in the research population could have contributed to these discrepancies. While some studies specifically focused on female patients, our analysis encompassed all patients, irrespective of gender. Second, unlike our study, the aforementioned research failed to consider the impact of ER, PR, and HER2 status on their interrelationships while adjusting for covariates. Furthermore, subgroup analysis was not conducted in the previous study. It is worth noting that the relationship between ER−/PR+ status and BC-LuC has not been definitively established in previous studies.

Our study sheds light on the potential impacts of ER, PR, and HER2 expression status on the overall and disease-specific survival of patients with BC-LuC. Specifically, we found that ER+ and PR+ statuses were associated with prolonged overall survival but decreased disease-specific survival in patients with BC-LuC. Furthermore, ER+, PR+, HER2+ statuses were also associated with prolonged OS but decreased DSS in BC patients. However, the results pertaining to HER2+ status were not entirely consistent. Previous studies [18–22] have reported a similar association between ER+ and PR+ status and improved OS, DSS, and progression-free survival, for both male and lactating breast cancer. This aligns with our findings, although we did not observe any correlation between ER−/PR+ status and clinical outcomes in BC-LuC due to the limited sample size. Our study's novel exploration of this breast cancer subtype warrants further investigation.

The present study involved a limited number of cases with metastasis in BC-LuC and aimed to analyze the role of ER-, -PR-, and HER2, as well as ER−/PR+ status, in both BC and BC-LuC overall. In line with Salvador's review [23], which examined breast cancer patients with distant disease, particularly those with the ER+ subtype showed a higher prevalence of bone metastasis. To identify breast cancer patients with brain metastases at diagnosis, a retrospective analysis was conducted using the Surveillance, Epidemiology, and End Results (SEER) database of the National Cancer Institute, including 238,726 adult patients diagnosed with invasive breast cancer between 2010 and 2013, with recorded information on the presence or absence of brain metastases [24]. The findings from this study indicated that HR−/HER2+ and triple-negative subtypes were more prone to brain metastasis. Consistently, Leone's study [25] also utilizing the SEER database, revealed that HR−/HER2+ subtype had higher odds of liver metastases, while triple-negative subtype had higher odds of brain metastases. These results corroborate our findings. Several studies [26, 27] have demonstrated that ER, PR, and HER2 status may change after breast cancer metastasis without significantly impacting progression-free survival. Given the close association between ER, PR, HER2 and metastasis, identifying indicators that are linked to an increased risk of metastasis is crucial. In this context, elucidating the relationship between ER−/PR+ status as an independent phenotype and metastasis holds particular significance for understanding the survival and burden of breast cancer patients.

The clinical significance of this study is twofold: first, it represents the pioneering observation of an independent association between ER−/PR+ status and the risk and outcomes in BC-LuC, specifically in the context of metastasis. To the best of our knowledge, this is the first study to report such an association. Second, the findings of this study hold considerable potential for guiding future research endeavors aimed at developing diagnostic or predictive models specifically tailored for BC-LuC. By shedding light on the relationship between ER−/PR+ status and BC-LuC, these findings serve as a valuable foundation for further investigations in this field.

This study is subject to several noteworthy limitations. First, the SEER database used in this study provided incomplete and limited information regarding smoking, alcohol consumption, and certain blood biochemical indicators associated with breast cancer prognosis. Consequently, the results of logistic and Cox analyses may have been affected. Second despite the large-scale nature of the patient cohort included in the SEER database, the number of eligible patients with metastasis for inclusion in this study remained relatively low, particularly within certain analytical categories. Therefore, it is imperative to validate our findings by including a larger number of patients from diverse geographical locations.

## 5. Conclusion

In conclusion, this study provides insights into the risk and outcomes of patients with BC-LuC. Our findings suggest that compared to patients with single breast cancer, the presence of PR+ and HER2+ subtypes may potentially be associated with a reduced risk of developing BC-LuC. Furthermore, we observed that ER+, PR+, HER2+, and ER−/PR+ status may have an impact on the outcomes of BC-LuC and BC patients. These observations highlight the need for further investigation into the association between molecular subtypes and BC-LuC. It is crucial to conduct additional clinical trials to confirm and validate these associations in order to enhance our understanding and clinical management of BC-LuC.

## Figures and Tables

**Table 1:** Baseline characteristics of participants.

Variables	ER				PR				HER2				ER−/PR+			
	Total (*n* = 198972)	Negative (*n* = 33335)	Positive (*n* = 165637)	*p*	Total (*n* = 198972)	Negative (*n* = 53490)	Positive (*n* = 145482)	*p*	Total (*n* = 198972)	Negative (*n* = 168484)	Positive (*n* = 30488)	*p*	Total (*n* = 198972)	Negative (*n* = 196890)	Positive (*n* = 2082)	*p*
Site recode (ICD.O.3), *n* (%)				0.254				0.195				<0.001				0.338
BC	196982 (99.0)	33021 (99.1)	163961 (99)		196982 (99.0)	52929 (99)	144053 (99)		196982 (99.0)	166720 (99)	30262 (99.3)		196982 (99.0)	194916 (99)	2066 (99.2)	
BC-LuC	1990 (1.0)	314 (0.9)	1676 (1)		1990 (1.0)	561 (1)	1429 (1)		1990 (1.0)	1764 (1)	226 (0.7)		1990 (1.0)	1974 (1)	16 (0.8)	
Age, *n* (%)				<0.001				<0.001				<0.001				<0.001
≤40	11337 (5.7)	3252 (9.8)	8085 (4.9)		11337 (5.7)	4267 (8)	7070 (4.9)		11337 (5.7)	8403 (5)	2934 (9.6)		11337 (5.7)	11120 (5.6)	217 (10.4)	
40–60	85657 (43.0)	16063 (48.2)	69594 (42)		85657 (43.0)	23738 (44.4)	61919 (42.6)		85657 (43.0)	70372 (41.8)	15285 (50.1)		85657 (43.0)	84617 (43)	1040 (50)	
≥60	101978 (51.3)	14020 (42.1)	87958 (53.1)		101978 (51.3)	25485 (47.6)	76493 (52.6)		101978 (51.3)	89709 (53.2)	12269 (40.2)		101978 (51.3)	101153 (51.4)	825 (39.6)	
Sex, *n* (%)				<0.001				<0.001				0.016				0.002
Male	1338 (0.7)	38 (0.1)	1300 (0.8)		1338 (0.7)	118 (0.2)	1220 (0.8)		1338 (0.7)	1165 (0.7)	173 (0.6)		1338 (0.7)	1336 (0.7)	2 (0.1)	
Female	197634 (99.3)	33297 (99.9)	164337 (99.2)		197634 (99.3)	53372 (99.8)	144262 (99.2)		197634 (99.3)	167319 (99.3)	30315 (99.4)		197634 (99.3)	195554 (99.3)	2080 (99.9)	
Race, *n* (%)				<0.001				<0.001				<0.001				<0.001
American Indian/Alaska native	1217 (0.6)	211 (0.6)	1006 (0.6)		1217 (0.6)	318 (0.6)	899 (0.6)		1217 (0.6)	991 (0.6)	226 (0.7)		1217 (0.6)	1198 (0.6)	19 (0.9)	
Asian or Pacific Islander	18801 (9.4)	3062 (9.2)	15739 (9.5)		18801 (9.4)	4969 (9.3)	13832 (9.5)		18801 (9.4)	15244 (9)	3557 (11.7)		18801 (9.4)	18616 (9.5)	185 (8.9)	
Black	20734 (10.4)	5851 (17.6)	14883 (9)		20734 (10.4)	8407 (15.7)	12327 (8.5)		20734 (10.4)	17070 (10.1)	3664 (12)		20734 (10.4)	20392 (10.4)	342 (16.4)	
White	158220 (79.5)	24211 (72.6)	134009 (80.9)		158220 (79.5)	39796 (74.4)	118424 (81.4)		158220 (79.5)	135179 (80.2)	23041 (75.6)		158220 (79.5)	156684 (79.6)	1536 (73.8)	
Marital status, *n* (%)				<0.001				<0.001				<0.001				0.017
Married	117748 (59.2)	19308 (57.9)	98440 (59.4)		117748 (59.2)	30739 (57.5)	87009 (59.8)		117748 (59.2)	99437 (59)	18311 (60.1)		117748 (59.2)	116518 (59.2)	1230 (59.1)	
Single	30858 (15.5)	5882 (17.6)	24976 (15.1)		30858 (15.5)	9047 (16.9)	21811 (15)		30858 (15.5)	25530 (15.2)	5328 (17.5)		30858 (15.5)	30494 (15.5)	364 (17.5)	
Divorced and separated	24532 (12.3)	4291 (12.9)	20241 (12.2)		24532 (12.3)	6863 (12.8)	17669 (12.1)		24532 (12.3)	20806 (12.3)	3726 (12.2)		24532 (12.3)	24278 (12.3)	254 (12.2)	
Widowed	25834 (13.0)	3854 (11.6)	21980 (13.3)		25834 (13.0)	6841 (12.8)	18993 (13.1)		25834 (13.0)	22711 (13.5)	3123 (10.2)		25834 (13.0)	25600 (13)	234 (11.2)	
Months from diagnosis to treatment, *n* (%)				<0.001				<0.001				<0.001				0.028
≤1 month	150419 (75.6)	26222 (78.7)	124197 (75)		150419 (75.6)	41654 (77.9)	108765 (74.8)		150419 (75.6)	127063 (75.4)	23356 (76.6)		150419 (75.6)	148793 (75.6)	1626 (78.1)	
1–3 months	44374 (22.3)	6443 (19.3)	37931 (22.9)		44374 (22.3)	10709 (20)	33665 (23.1)		44374 (22.3)	37894 (22.5)	6480 (21.3)		44374 (22.3)	43957 (22.3)	417 (20)	
>3 months	4179 (2.1)	670 (2)	3509 (2.1)		4179 (2.1)	1127 (2.1)	3052 (2.1)		4179 (2.1)	3527 (2.1)	652 (2.1)		4179 (2.1)	4140 (2.1)	39 (1.9)	
CS tumor size, *n* (%)				<0.001				<0.001				<0.001				<0.001
≤2 cm	110122 (55.3)	12834 (38.5)	97288 (58.7)		110122 (55.3)	22907 (42.8)	87215 (59.9)		110122 (55.3)	97003 (57.6)	13119 (43)		110122 (55.3)	109346 (55.5)	776 (37.3)	
2–5 cm	70660 (35.5)	15520 (46.6)	55140 (33.3)		70660 (35.5)	23168 (43.3)	47492 (32.6)		70660 (35.5)	57443 (34.1)	13217 (43.4)		70660 (35.5)	69635 (35.4)	1025 (49.2)	
>5 cm	18190 (9.1)	4981 (14.9)	13209 (8)		18190 (9.1)	7415 (13.9)	10775 (7.4)		18190 (9.1)	14038 (8.3)	4152 (13.6)		18190 (9.1)	17909 (9.1)	281 (13.5)	
Laterality, *n* (%)				0.126				0.004				0.002				0.108
Left	101188 (50.9)	17122 (51.4)	84066 (50.8)		101188 (50.9)	27530 (51.5)	73658 (50.6)		101188 (50.9)	85477 (50.7)	15711 (51.5)		101188 (50.9)	100157 (50.9)	1031 (49.5)	
Right	97759 (49.1)	16209 (48.6)	81550 (49.2)		97759 (49.1)	25953 (48.5)	71806 (49.4)		97759 (49.1)	82990 (49.3)	14769 (48.4)		97759 (49.1)	96709 (49.1)	1050 (50.4)	
Bilateral	25 (0.0)	4 (0)	21 (0)		25 (0.0)	7 (0)	18 (0)		25 (0.0)	17 (0)	8 (0)		25 (0.0)	24 (0)	1 (0)	
Combined summary stage, *n* (%)				<0.001				<0.001				<0.001				<0.001
Distant	8249 (4.1)	2091 (6.3)	6158 (3.7)		8249 (4.1)	3298 (6.2)	4951 (3.4)		8249 (4.1)	6028 (3.6)	2221 (7.3)		8249 (4.1)	8119 (4.1)	130 (6.2)	
Localized	129366 (65.0)	19351 (58.1)	110015 (66.4)		129366 (65.0)	31682 (59.2)	97684 (67.1)		129366 (65.0)	112971 (67.1)	16395 (53.8)		129366 (65.0)	128148 (65.1)	1218 (58.5)	
Regional	61357 (30.8)	11893 (35.7)	49464 (29.9)		61357 (30.8)	18510 (34.6)	42847 (29.5)		61357 (30.8)	49485 (29.4)	11872 (38.9)		61357 (30.8)	60623 (30.8)	734 (35.3)	
AJCC (7th), *n* (%)				<0.001				<0.001				<0.001				<0.001
I	100449 (50.5)	11383 (34.1)	89066 (53.8)		100449 (50.5)	20480 (38.3)	79969 (55)		100449 (50.5)	89243 (53)	11206 (36.8)		100449 (50.5)	99758 (50.7)	691 (33.2)	
II	68478 (34.4)	14611 (43.8)	53867 (32.5)		68478 (34.4)	21714 (40.6)	46764 (32.1)		68478 (34.4)	56275 (33.4)	12203 (40)		68478 (34.4)	67505 (34.3)	973 (46.7)	
III	22143 (11.1)	5409 (16.2)	16734 (10.1)		22143 (11.1)	8218 (15.4)	13925 (9.6)		22143 (11.1)	17150 (10.2)	4993 (16.4)		22143 (11.1)	21848 (11.1)	295 (14.2)	
IV	7902 (4.0)	1932 (5.8)	5970 (3.6)		7902 (4.0)	3078 (5.8)	4824 (3.3)		7902 (4.0)	5816 (3.5)	2086 (6.8)		7902 (4.0)	7779 (4)	123 (5.9)	
Radiation, *n* (%)				<0.001				<0.001				<0.001				<0.001
Without	86484 (43.5)	16061 (48.2)	70423 (42.5)		86484 (43.5)	25168 (47.1)	61316 (42.1)		86484 (43.5)	71351 (42.3)	15133 (49.6)		86484 (43.5)	85499 (43.4)	985 (47.3)	
With	112488 (56.5)	17274 (51.8)	95214 (57.5)		112488 (56.5)	28322 (52.9)	84166 (57.9)		112488 (56.5)	97133 (57.7)	15355 (50.4)		112488 (56.5)	111391 (56.6)	1097 (52.7)	
Chemotherapy, *n* (%)				<0.001				<0.001				<0.001				<0.001
Without	113170 (56.9)	7292 (21.9)	105878 (63.9)		113170 (56.9)	17163 (32.1)	96007 (66)		113170 (56.9)	106248 (63.1)	6922 (22.7)		113170 (56.9)	112673 (57.2)	497 (23.9)	
With	85802 (43.1)	26043 (78.1)	59759 (36.1)		85802 (43.1)	36327 (67.9)	49475 (34)		85802 (43.1)	62236 (36.9)	23566 (77.3)		85802 (43.1)	84217 (42.8)	1585 (76.1)	
Disease-specific death, *n* (%)				<0.001				<0.001				<0.001				<0.001
Alive	179397 (90.2)	26783 (80.3)	152614 (92.1)		179397 (90.2)	44141 (82.5)	135256 (93)		179397 (90.2)	152477 (90.5)	26920 (88.3)		179397 (90.2)	177724 (90.3)	1673 (80.4)	
Death	19575 (9.8)	6552 (19.7)	13023 (7.9)		19575 (9.8)	9349 (17.5)	10226 (7)		19575 (9.8)	16007 (9.5)	3568 (11.7)		19575 (9.8)	19166 (9.7)	409 (19.6)	
Survival months (mean ± SD)	71.8 ± 27.6	66.1 ± 31.4	73.0 ± 26.7	<0.001	71.8 ± 27.6	67.5 ± 30.6	73.4 ± 26.3	<0.001	71.8 ± 27.6	72.1 ± 27.6	70.5 ± 28.1	<0.001	71.8 ± 27.6	71.9 ± 27.6	66.7 ± 31.0	<0.001
All-cause death, *n* (%)				<0.001				<0.001				0.652				<0.001
Alive	164475 (82.7)	24551 (73.6)	139924 (84.5)		164475 (82.7)	40176 (75.1)	124299 (85.4)		164475 (82.7)	139245 (82.6)	25230 (82.8)		164475 (82.7)	162922 (82.7)	1553 (74.6)	
Death	34497 (17.3)	8784 (26.4)	25713 (15.5)		34497 (17.3)	13314 (24.9)	21183 (14.6)		34497 (17.3)	29239 (17.4)	5258 (17.2)		34497 (17.3)	33968 (17.3)	529 (25.4)	
Bone metastasis, *n* (%)				0.265				<0.001				<0.001				0.853
Without	193730 (97.4)	32487 (97.5)	161243 (97.3)		193730 (97.4)	51865 (97)	141865 (97.5)		193730 (97.4)	164456 (97.6)	29274 (96)		193730 (97.4)	191701 (97.4)	2029 (97.5)	
With	5242 (2.6)	848 (2.5)	4394 (2.7)		5242 (2.6)	1625 (3)	3617 (2.5)		5242 (2.6)	4028 (2.4)	1214 (4)		5242 (2.6)	5189 (2.6)	53 (2.5)	
Brain metastasis, *n* (%)				<0.001				<0.001				<0.001				0.282
Without	198408 (99.7)	33129 (99.4)	165279 (99.8)		198408 (99.7)	53184 (99.4)	145224 (99.8)		198408 (99.7)	168091 (99.8)	30317 (99.4)		198408 (99.7)	196335 (99.7)	2073 (99.6)	
With	564 (0.3)	206 (0.6)	358 (0.2)		564 (0.3)	306 (0.6)	258 (0.2)		564 (0.3)	393 (0.2)	171 (0.6)		564 (0.3)	555 (0.3)	9 (0.4)	
Liver metastasis, *n* (%)				<0.001				<0.001				<0.001				<0.001
Without	196984 (99.0)	32668 (98)	164316 (99.2)		196984 (99.0)	52524 (98.2)	144460 (99.3)		196984 (99.0)	167307 (99.3)	29677 (97.3)		196984 (99.0)	194950 (99)	2034 (97.7)	
With	1988 (1.0)	667 (2)	1321 (0.8)		1988 (1.0)	966 (1.8)	1022 (0.7)		1988 (1.0)	1177 (0.7)	811 (2.7)		1988 (1.0)	1940 (1)	48 (2.3)	
Lung metastasis, *n* (%)				<0.001				<0.001				<0.001				<0.001
Without	196625 (98.8)	32585 (97.8)	164040 (99)		196625 (98.8)	52444 (98)	144181 (99.1)		196625 (98.8)	166805 (99)	29820 (97.8)		196625 (98.8)	194596 (98.8)	2029 (97.5)	
With	2347 (1.2)	750 (2.2)	1597 (1)		2347 (1.2)	1046 (2)	1301 (0.9)		2347 (1.2)	1679 (1)	668 (2.2)		2347 (1.2)	2294 (1.2)	53 (2.5)	

*Note*. BC-LuC: breast cancer with primary lung cancer; BC: breast cancer; ER: estrogen receptor; PR: progesterone receptor; HER2: human epidermal growth factor receptor 2. *p* value <0.05 was considered statistically significant.

**Table 2:** Multivariable-adjust OR and 95% CI of the ER, PR, HER2, and ER−/PR+ associated with BC-LuC.

	PR+			ER+			HER2+			ER−/PR+		
	*N*(%)	OR (95% CI)	*p*value	*N*(%)	OR (95% CI)	*p*value	*N*(%)	OR (95% CI)	*p*value	*N*(%)	OR (95% CI)	*p*value
Unadjusted model	1429 (1.0)	0.94 (0.85∼1.03)	0.186	1676 (1.0)	1.07 (0.95∼1.21)	0.242	226 (0.7)	0.71 (0.61∼0.81)	<0.001	16 (0.8)	0.76 (0.47∼1.25)	0.623
Model 1	1429 (1.0)	0.88 (0.79∼0.97)	0.009	1676 (1.0)	0.93 (0.83∼1.05)	0.264	226 (0.7)	0.83 (0.72∼0.95)	0.008	16 (0.8)	0.89 (0.54∼1.46)	0.644
Model 2	1429 (1.0)	0.88 (0.80∼0.97)	0.011	1676 (1.0)	0.94 (0.83∼1.06)	0.304	226 (0.7)	0.83 (0.72∼0.96)	0.011	16 (0.8)	0.88 (0.54∼1.45)	0.623
Model 3	1429 (1.0)	0.84 (0.75∼0.93)	0.001	1676 (1.0)	0.89 (0.78∼1.02)	0.09	226 (0.7)	0.85 (0.74∼0.99)	0.034	16 (0.8)	0.91 (0.55∼1.49)	0.694
Model 4	1429 (1.0)	0.81 (0.71∼0.93)	0.002	1676 (1.0)	1.03 (0.87∼1.22)	0.718	226 (0.7)	0.83 (0.72∼0.96)	0.012	16 (0.8)	1.02 (0.61∼1.72)	0.936

Adjusted covariates: model 1 = age and sex. Model 2 = model 1+ (race, laterality, martial, and month from diagnosis treatment). Model 3 = model 2+ (AJCC, stage, CS tumor size, radiation, and chemotherapy). Model 4 = model 3+ (ER, PR, HER2, and breast subtype).

## Data Availability

The data used to support the findings of this study are available from the corresponding author on reasonable request.

## Supplementary Materials

Supplementary Materials Supplementary Table 1: multivariable Cox regression analyses HR and 95% CI of the ER, PR, HER2, and ER−/PR+ associated with outcomes in BC-LuC. Supplementary Table 2: multivariable Cox regression analyses HR and 95% CI of the ER, PR, HER2, and ER−/PR+ associated with outcomes in BC. Supplementary Table 3: the association of OR and 95% CI between the ER, PR, HER2, and ER−/PR+ with metastasis in BC and BC-LuC. Supplementary Figure 1: Kaplan–Meier survival curves for DSS of ER, PR, and HER2 and ER−/PR+ in BC. Supplementary Figure 2: Kaplan–Meier survival curves for OS of ER, PR, and HER2 and ER−/PR+ in BC.
