# Supplementary material for: The Association between ER, PR, HER2, and ER−/PR+ Expression and Lung Cancer Subsequent in Breast Cancer Patients: A Retrospective Cohort Study Based on SEER Database
Source: Breast J. 2023 Nov 11;2023:7028189. doi: 10.1155/2023/7028189 (PMC10657240; doi:10.1155/2023/7028189)

**Supplementary Table1** Multi-variable Cox regression analyses HR and 95%CI of the ER，PR,HER2,ER-/PR+ associated with outcomes in BC-LuC.

| Variable | Outcomes | N（%） | HR(95%CI) | P-value | HR(95%CI) | P-value |
| --- | --- | --- | --- | --- | --- | --- |
| ER+ | Disease specific survival | 211(12.6) | 0.51 (0.39~0.68) | <0.001 | 0.72 (0.48~1.06) | 0.095 |
|  | Overall survival | 910 (54.3) | 0.74(0.63~0.86) | <0.001 | 0.67 (0.54~0.83) | <0.001 |
| PR+ | Disease specific survival | 170 (11.9) | 0.57 (0.44~0.72) | <0.001 | 0.58(0.42~0.82) | 0.002 |
|  | Overall survival | 772 (54) | 0.82 (0.72~0.93) | 0.002 | 0.85 (0.71~1.02) | 0.086 |
| HER+ | Disease specific survival | 32(14.2) | 1.02(0.71~1.48) | 0.912 | 0.64 (0.43~0.95) | 0.028 |
|  | Overall survival | 115(50.9) | 0.91 (0.75~1.11) | 0.351 | 0.87 (0.71~1.06) | 0.165 |
| ER-/PR+ | Disease specific survival | 5(31.2) | 2.3(0.95~5.58) | 0.064 | 2.43 (0.88~6.74) | 0.087 |
|  | Overall survival | 8 (50) | 0.91 (0.45~1.82) | 0.783 | 0.90(0.43~1.90) | 0.788 |

**Supplementary Table2** Multi-variable Cox regression analyses HR and 95%CI of the ER，PR,HER2,ER-/PR+ associated with outcomes in BC.

| Variable | Outcomes | N（%） | HR(95%CI) | *P*-value | HR(95%CI) | *P*-value |
| --- | --- | --- | --- | --- | --- | --- |
| ER+ | Disease specific survival | 12812 (7.8) | 0.36 (0.35~0.37) | <0.001 | 0.66 (0.63~0.69) | <0.001 |
|  | Overall survival | 24803 (15.1) | 0.53 (0.51~0.54) | <0.001 | 0.68 (0.66~0.71) | <0.001 |
| PR+ | Disease specific survival | 10056 (7) | 0.37 (0.36~0.38) | <0.001 | 0.53 (0.51~0.55) | <0.001 |
|  | Overall survival | 20411 (14.2) | 0.53 (0.52~0.54) | <0.001 | 0.65 (0.63~0.67) | <0.001 |
| HER+ | Disease specific survival | 3536 (11.7) | 1.26 (1.22~1.31) | <0.001 | 0.63 (0.6~0.65) | <0.001 |
|  | Overall survival | 5143 (17) | 1.03 (1~1.06) | 0.093 | 0.74 (0.71~0.76) | <0.001 |
| ER-/PR+ | Disease specific survival | 404 (19.6) | 2.17 (1.97~2.4) | <0.001 | 1.86 (1.67~2.08) | <0.001 |
|  | Overall survival | 521 (25.2) | 1.62 (1.48~1.76) | <0.001 | 1.47 (1.33~1.61) | <0.001 |

**Supplementary Table3** The association of OR and 95%CI bteween the ER，PR,HER2,ER-/PR+ with metastasis in BC and BC-LuC.

| Location | Variable | N（%）） | OR（95%CI） | P-value | OR（95%CI） | P-value |
| --- | --- | --- | --- | --- | --- | --- |
| Bone | ER+ | 4394 (2.7) | 1.04 (0.97~1.12) | 0.257 | 2.53 (2.2~2.91) | <0.001 |
|  | PR+ | 3617 (2.5) | 0.81 (0.77~0.86) | <0.001 | 1.49 (1.31~1.7) | <0.001 |
|  | HER2+ | 1214 (4) | 1.69 (1.59~1.81) | <0.001 | 0.82 (0.74~0.92) | 0.001 |
|  | ER-/PR+ | 53 (2.5) | 0.97 (0.73~1.27) | 0.799 | 0.65 (0.43~0.96) | 0.032 |
| Lung | ER+ | 1597 (1) | 0.42 (0.39~0.46) | <0.001 | 0.57 (0.5~0.67) | <0.001 |
|  | PR+ | 1301 (0.9) | 0.45 (0.42~0.49) | <0.001 | 1.03 (0.9~1.18) | 0.675 |
|  | HER2+ | 668 (2.2) | 2.23 (2.03~2.44) | <0.001 | 1.11 (0.99~1.24) | 0.086 |
|  | ER-/PR+ | 53 (2.5) | 2.22 (1.68~2.92) | <0.001 | 1.14 (0.76~1.71) | 0.517 |
|  | ER+ | 1321 (0.8) | 0.39 (0.36~0.43) | <0.001 | 0.74 (0.64~0.87) | <0.001 |
| Liver | PR+ | 1022 (0.7) | 0.38 (0.35~0.42) | <0.001 | 0.84 (0.73~0.96) | 0.012 |
|  | HER2+ | 811 (2.7) | 3.88 (3.55~4.25) | <0.001 | 1.98 (1.76~2.22) | <0.001 |
|  | ER-/PR+ | 48 (2.3) | 2.37 (1.78~3.17) | <0.001 | 1.58 (1.05~2.4) | 0.03 |
|  | ER+ | 358 (0.2) | 0.35 (0.29~0.41) | <0.001 | 0.69 (0.54~0.87) | 0.002 |
| Brain | PR+ | 258 (0.2) | 0.31 (0.26~0.36) | <0.001 | 0.6 (0.48~0.76) | <0.001 |
|  | HER2+ | 171 (0.6) | 2.41 (2.02~2.89) | <0.001 | 1.13 (0.92~1.38) | 0.247 |
|  | ER-/PR+ | 9 (0.4) | 1.54 (0.79~2.97) | 0.203 | 1.27 (0.59~2.71) | 0.538 |
|  |  |  |  |  |  |  |

**Supplementary Figure 1 Kaplan-Meier Survival Curves for DSS of ER, PR, HER2 and ER-PR+ in BC.**


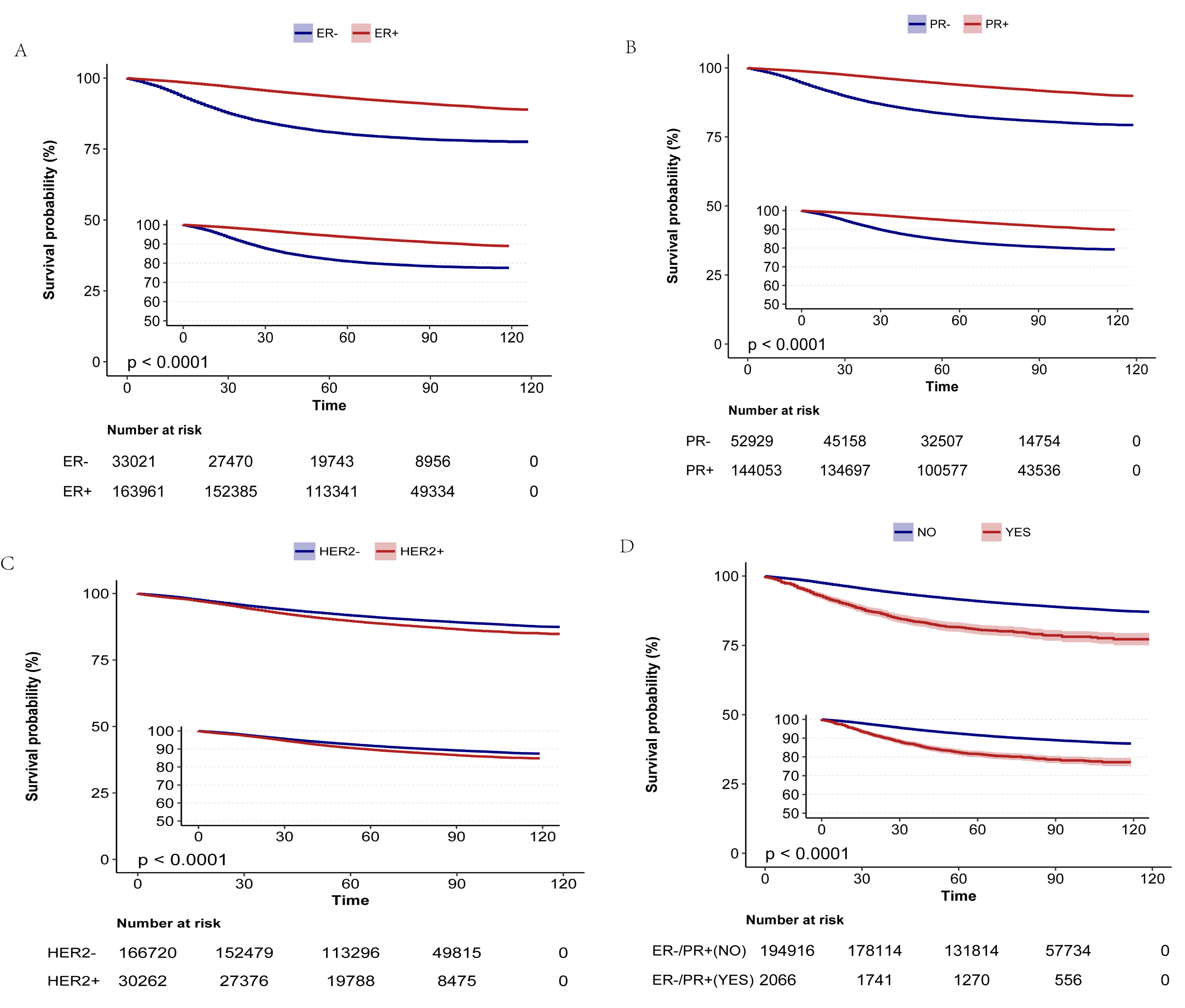


**Supplementary Figure2 Kaplan-Meier Survival Curves for OS of ER, PR, HER2 and ER-PR+ in BC.**


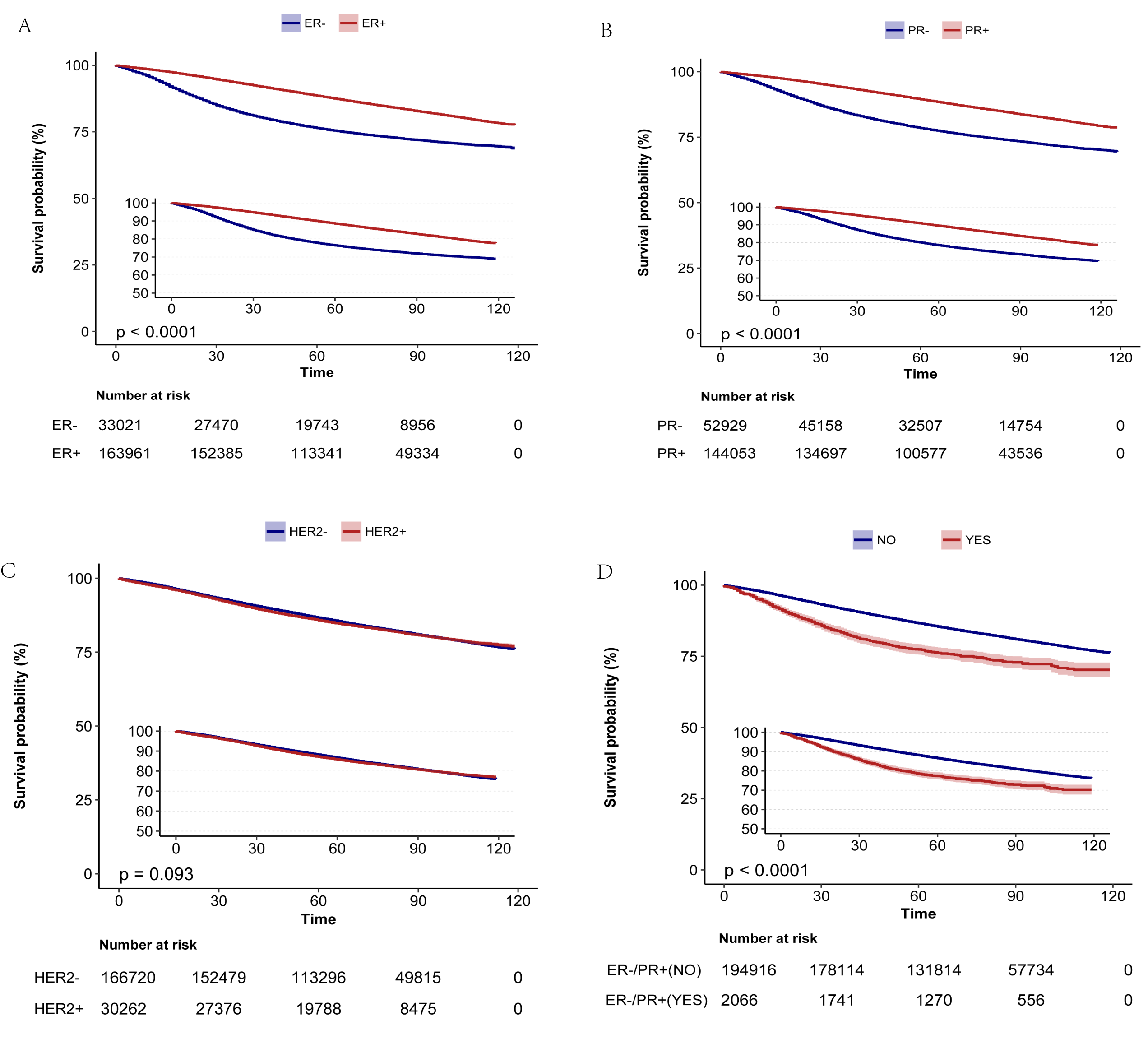

Supplement: Supplementary Materials — Supplementary Table 1: multivariable Cox regression analyses HR and 95% CI of the ER, PR, HER2, and ER−/PR+ associated with outcomes in BC-LuC. Supplementary Table 2: multivariable Cox regression analyses HR and 95% CI of the ER, PR, HER2, and ER−/PR+ associated with outcomes in BC. Supplementary Table 3: the association of OR and 95% CI between the ER, PR, HER2, and ER−/PR+ with metastasis in BC and BC-LuC. Supplementary Figure 1: Kaplan–Meier survival curves for DSS of ER, PR, and HER2 and ER−/PR+ in BC. Supplementary Figure 2: Kaplan–Meier survival curves for OS of ER, PR, and HER2 and ER−/PR+ in BC. [file 7028189.f1.zip › supplementary material.docx]
